# A systematic review and meta-analysis of proteomic and metabolomic alterations in anaphylaxis reactions

**DOI:** 10.3389/fimmu.2024.1328212

**Published:** 2024-02-07

**Authors:** Adrienne Astrid Gallizzi, Almut Heinken, Rosa-Maria Guéant-Rodriguez, Jean-Louis Guéant, Ramia Safar

**Affiliations:** ^1^INSERM, UMR_S1256, NGERE – Nutrition, Genetics, and Environmental Risk Exposure, Faculty of Medicine of Nancy, University of Lorraine, Vandoeuvre-lès-Nancy, France; ^2^Department of Molecular Medicine and Personalized Therapeutics, Department of Biochemistry, Molecular Biology, Nutrition, and Metabolism, University Hospital of Nancy, Vandoeuvre-lès-Nancy, France

**Keywords:** anaphylaxis, omics studies, proteome, metabolome, neutrophil, platelets, arachidonic acid, icosatetraenoic acid

## Abstract

**Background:**

Anaphylaxis manifests as a severe immediate-type hypersensitivity reaction initiated through the immunological activation of target B-cells by allergens, leading to the release of mediators. However, the well-known underlying pathological mechanisms do not fully explain the whole variety of clinical and immunological presentations. We performed a systemic review of proteomic and metabolomic studies and analyzed the extracted data to improve our understanding and identify potential new biomarkers of anaphylaxis.

**Methods:**

Proteomic and metabolomic studies in both human subjects and experimental models were extracted and selected through a systematic search conducted on databases such as PubMed, Scopus, and Web of Science, up to May 2023.

**Results:**

Of 137 retrieved publications, we considered 12 for further analysis, including seven on proteome analysis and five on metabolome analysis. A meta-analysis of the four human studies identified 118 proteins with varying expression levels in at least two studies. Beside established pathways of mast cells and basophil activation, functional analysis of proteomic data revealed a significant enrichment of biological processes related to neutrophil activation and platelet degranulation and metabolic pathways of arachidonic acid and icosatetraenoic acid. The pathway analysis highlighted also the involvement of neutrophil degranulation, and platelet activation. Metabolome analysis across different models showed 13 common metabolites, including arachidonic acid, tryptophan and lysoPC(18:0) lysophosphatidylcholines.

**Conclusion:**

Our review highlights the underestimated role of neutrophils and platelets in the pathological mechanisms of anaphylactic reactions. These findings, derived from a limited number of publications, necessitate confirmation through human studies with larger sample sizes and could contribute to the development of new biomarkers for anaphylaxis.

**Systematic review registration:**

https://www.crd.york.ac.uk/PROSPERO/, identifier CRD42024506246.

## 1 Introduction

Anaphylaxis is generally considered to be the most severe manifestation of immediate hypersensitivity reactions, induced by the introduction of an allergen into the body. The exact definition of anaphylaxis and the criteria for its diagnosis remain to be subjects of ongoing debate within the scientific community (1, 2). It is an immune response (immunoglobulin E [IgE]- or non-IgE-dependent) leading to the release of mediators in target cells, especially mast cells and basophils. The term “anaphylactoid reaction” has been used to describe clinically similar outcomes produced by a non-immune response to certain antigens (3).

The exact frequency of anaphylaxis is not well-established and is often underestimated, but it appears to be increasing. Over the last decade, the incidence of hospital admissions for anaphylaxis has surged, reaching up to sevenfold in recent years (4). Prevalence depends on geographic location, gender, atopy, and socioeconomic factors (5). Drugs constitute the primary cause of anaphylaxis, with an estimated mortality rate of approximately 0.5-0.51 per million person/year, as indicated by the most recent World Allergy Organization (WAO) anaphylaxis guidance published in 2020 (6). Despite the low rate of mortality, the percentage of fatal anaphylaxis cases due to drugs is estimated around 60% of total fatal anaphylaxis cases (7, 8). The principal drugs responsible for anaphylaxis are the antibiotics including cephalosporins and penicillin, neuromuscular-blocking agents (NMBAs), non-steroidal anti-inflammatory (NSAIDs), injectable radiocontrast agents, therapeutic antibodies, and antineoplastic agents (7–9).

The severity of an anaphylactic event can be assessed using a scoring system known as the Brown severity scale. This scale consists of three levels: mild, rarely fatal, characterized by mild symptoms such as generalized erythema and urticaria; moderate, which can be life-threatening if left untreated, with major symptoms including dyspnea, sweating, and chest or throat tightness; severe, with a high risk of mortality due to the potential development of severe complications or organ failure, major symptoms being cyanosis, hypotension, and confusion (10). The variety and systemic manifestations of anaphylaxis reflects the diversity of mediators and effector cells implicated in the reaction, although some of activated pathways may overlap. Despite the varied outcomes and mechanisms, the diagnosis of anaphylaxis usually depends on the measurement of histamine and serum tryptase during the reaction. However, these biomarkers may not be altered in all cases, prompting a reconsideration and exploration of alternative biomarkers (11). The anaphylactic reaction results predominantly from an IgE-mediated release mediators initiated by the interaction between allergens, typically proteins, peptides or haptenized drug and chemicals, and allergen-specific IgE bound to high-affinity receptors (FcϵRI) on effector cells, primarily basophils and mast cells (12). Indeed, mast cells play a crucial role in the initiation of anaphylaxis (13). Cases with “mastocytosis” (high number of mastocytes) have a high incidence of anaphylaxis (14). The interaction allergen-receptor led to the release of mediators preformed and new synthesized (15). The preformed mediators include histamine, heparin, tryptase, chymase, tumor necrosis factor-alpha (TNFα), and cathepsin G. Newly formed mediators include platelet-activating factor (PAF), prostaglandin D2 (PGD2), leukotriene C4, cytokines and GM-CSF, as well as chemokines (16). While IgE-mediated release of mediators is the predominant mechanism of anaphylaxis, other non-IgE-mediated mechanisms of target cell activation and other target cells such as platelets and eosinophils have been documented (17–19). Which specific receptor is activated in which effector cells determines the type, location and magnitude of mediator release, and consequently, the type and severity of manifestations are still issues which need to be better evaluated (20).

It is very challenging to improve our knowledge on the immunologic mechanisms of anaphylaxis in humans. Predicting the onset of an anaphylactic reaction is not straightforward, and there are only a few clinical studies due to ethical concerns on provoking anaphylaxis. In the past several years, high-throughput multi-omics approaches have been developed to identify potential biomarkers of anaphylaxis, including genes, transcripts, proteins, and metabolites. However, the already known mechanisms do not fully explain the whole variety of clinical and immunological presentation. This suggests that, in addition to IgE-mediated reactions involving mast cells and basophils, other target cells and mediators likely contribute significantly in the reaction. A recent position paper in 2022, the EAACI Task Force “Omics technologies in allergic research”, has discussed the applicability of omics and the advances of these techniques in the allergy research (21). These high-performance omics technologies can allow to understand better the mechanisms and provide molecular signatures of anaphylaxis (22). Despite this evidence, only a restricted number of omics studies have been published in recent decades. The objective of our work was to conduct a systematic review of the proteomic and metabolomic studies published thus far, aiming to identify potential new cellular targets, mediators, and mechanisms of cell activation and release. This approach seeks to enhance our understanding and identify potential new biomarkers for anaphylaxis.

## 2 Materials and methods

### 2.1 Eligibility criteria

A literature search was performed up to May 2023 using keywords “Proteome and Anaphylaxis” and “Metabolome and Anaphylaxis.” The inclusion criteria comprised: 1) original articles published in English; 2) studies on patients experiencing anaphylaxis; or experimental mechanistic studies using animal models in an anaphylaxis context; 3) analyses of proteome or metabolome performed on various types of samples. Exclusion criteria included: 1) review papers and publications in languages other than English; 2) absence of data on proteome or metabolome, and data not exclusively focused on the moment of anaphylaxis.

### 2.2 Data extraction

Data extraction followed an organized structure, including the following information when available: author, year, geographical region, sample type, phenotype and number of cases, phenotype and number of controls, primary aim, study method and main finding. Two investigators (AAG, RS) conducted separate assessments of the titles and abstracts for all citations found during the literature search. Any disagreements were settled through consensus.

### 2.3 Functional and pathways enrichment analyses

We performed an enrichment analysis on proteins showing differential expression levels related to anaphylaxis using Flame tool (v2.0) (23). We included data from all human (patients and *in vitro*) studies and excluded data from animal model studies. In our biological interpretation, we considered the outputs with a *P-*value < 0.05 in the categories of gene ontology biological process (GO-BP), gene ontology molecular function (GO-MF), and pathway terms (KEGG and REACTOME).

## 3 Results

### 3.1 Anaphylaxis and proteome

#### 3.1.1 Literature search findings

**Figure 1:**
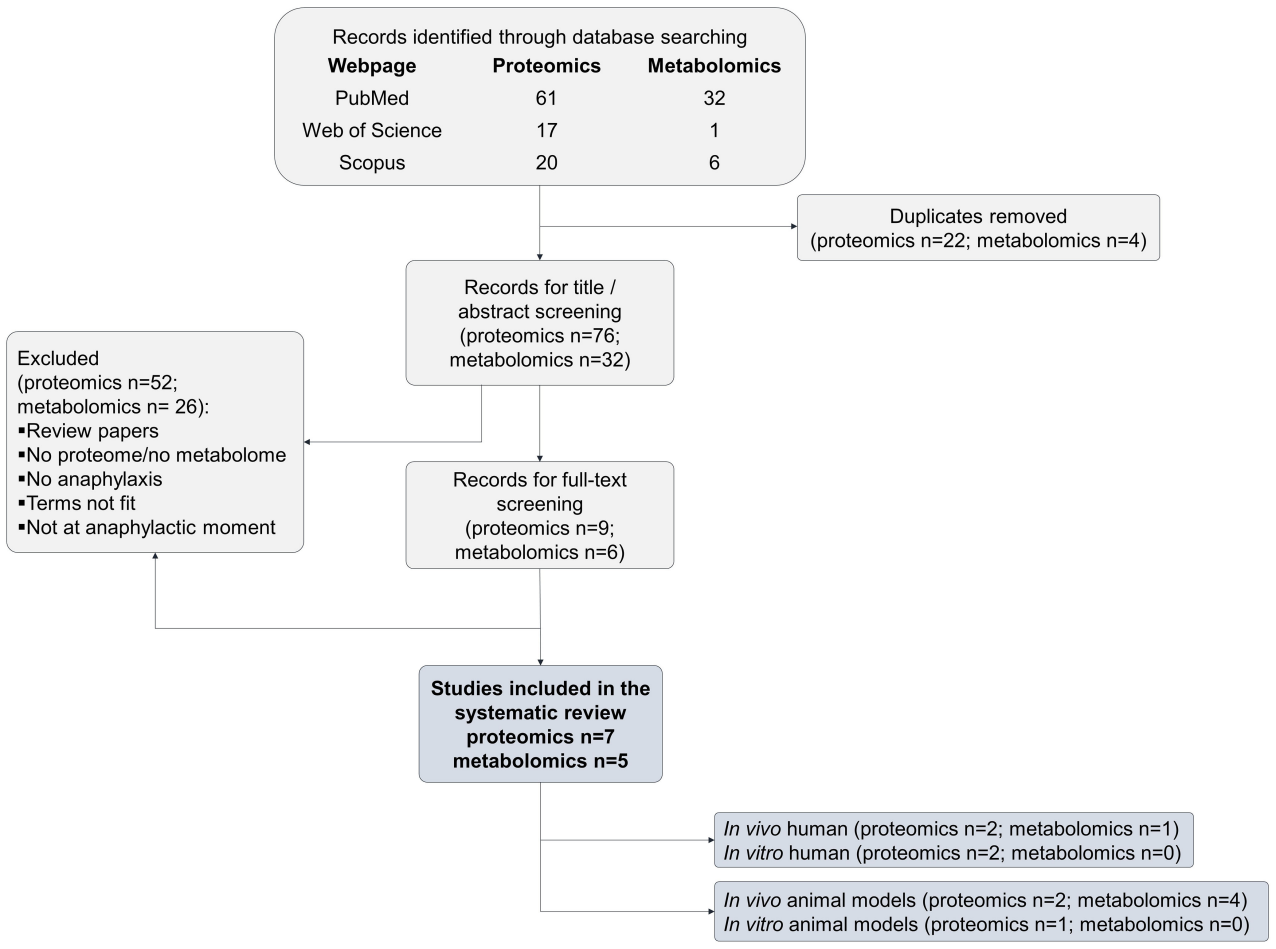
PRISMA flow diagram of the selection process for eligible articles in the systematic review.

The first search approach “anaphylaxis and proteome” yielded 98 citations. After excluding 91 studies, seven remained eligible for inclusion in this review (24–30). (**Figure 1** and **Supplementary Table 1**). Among them, four assessed the proteome in human studies (24–27) and three evaluated the proteome using an animal model (28–30) (**Table 1**).

**Table 1:** Studies that evaluated proteome alteration in anaphylactic condition.

First author Year	Geographical region	Sample type	Phenotype	Cases (n°)	Controls (n°)	Primary aim	Proteomics study method	Main finding	Ref.
*in vitro* human studies									
**Yuste-Montalvo, A.** **2021**	Spain	Endothelial cells (ECs)	Anaphylactic patients, drugs (antibiotics or NSAID)	–	–	To study ECs cells behavior when contact with serum from anaphylactic patients and control through a proteomic approach.	LC-MS/MS, nanoHPLC chromatography coupled to an Orbitrap Fusion Tribrid mass spectrometer	A total of 1,069 proteins exhibited significant alterations between the cell-control and cell-anaphylaxis groups. Among them, a sub-proteome consisting of 47 proteins (38 increased and 9 decreased) demonstrated a substantial degree of change.	(26)
**Yan-Ni, M.** **2020**	China	Laboratory of Allergic Diseases 2 (LAD2) mast cells	Tween-80 (polysorbate 80) -other-	–	–	To understand the mechanism of Tween-80 induced anaphylactoid reaction.	LC-MS/MS analysis (HPLC and Q-Exactive HF mass spectrometer).	313 up-regulated and 111 down-regulated proteins were found in Tween-80 group. These proteins are implicated in cellular process, biological regulation, and metabolic process and in several KEGG pathways such as endocytosis, cell adhesion molecules (CAMs), NF-κB signaling pathway and calcium signaling pathway.	(27)
*in vivo* human studies									
**Gulen, T.** **2021**	Sweden	Plasma	Mastocytosis	11 allergic asthma patients	13 healthy patients	To pinpoint specific biomarkers in plasma for predicting individuals with mastocytosis at a heightened risk of experiencing anaphylaxis.	Protein Extension Assay (PEA), real-time PCR	4 proteins were identified with significant difference between patients with mastocytosis with or without anaphylaxis: galectin-3/Gal-3 was upregulated and allergin-1/MILR1, tryptase/TPSAB1 and pregnancy-associated plasma protein-A/PAPPA were downregulated.	(24)
**Nuñez-Borque, E.** **2021**	Spain	Plasma	Drug, food, others	43 patients during the acute phase of anaphylaxis	43 same patients at the baseline	To assessed the use of extracellular vesicles (EV) as a potential marker of anaphylaxis.	Mass spectrometry	99 differentially expressed extracellular vesicle proteins were identified when compared the acute phase with the baseline proteins. 83 of them were up-regulated and may be serve as potential biomarkers.	(25)
*in vitro* animal model studies									
**Hao, F.** **2022**	China	RBL-2H3 cell line/rat	Panax notoginseng saponin (PNS)	–	–	To pinpoint the specific anaphylactic components in PNS and to clarify the underlying mechanism.	Liquid chroma- tography, HPLC, preparative chromatography, ESI-MS and NMR.	Four downregulated proteins were identified in the differentially expressed proteins of the strong anaphylactic groups. They are (Gtf2h1), (Hccs), (Pdha1l1), and Q8K3F0 (UniProtKB unreviewed/TrEMBL).	(30)
*in vivo* animal model studies									
**Yubin, X.** **2015**	China	Plasma	Xuesaitong injection (XSTI)	n=8 and n=8 (biological replicate)	n=8 and n=8 (biological replicate)	To evaluated the anaphylactoid reaction of Xuesaitong injection, an herbal medicine.	Mass spectrometry	143 differential proteins are identified in the XSTI group. Of which, F13b, F12, Kng1, C2 and C6 are involved in complement and coagulation pathways.	(28)
**Hobson, D.J.** **2007**	Canada	Plasma	Food, ovomucoid	n=15 female, BALB/c mice	n=15 female, BALB/c mice	To identify protein biomarkers associated with food allergy in mice exposed to ovomucoid.	Two-dimensional difference gel electrophoresis	Three proteins, Hp, SAA2, and PrxII, are found as upregulated in the plasma of ovomucoid -sensitized mice during induced anaphylaxis.	(29)

*1. Human studies:*

Two studies assessed the proteome change in anaphylaxis in patients (24, 25). In the first study, the authors aimed to pinpoint specific biomarkers in plasma for predicting individuals with mastocytosis at a heightened risk of experiencing anaphylaxis. The study included 19 patients, with 11 allergic asthma patients as the control group and 13 volunteers without atopy. Using Proximity Extension Assay with Olink Proseek Multiplex panels, a total of 248 plasma proteins were analyzed. Among them, four proteins showed significant differences between patients with mastocytosis and anaphylaxis compared to those without anaphylaxis. Specifically, galectin-3 (Gal-3) was upregulated, while allergin-1 (MILR1), tryptase (TPSAB1), and pregnancy-associated plasma protein-A (PAPPA) were downregulated (24). In the second study conducted by Nunez-Borque et al. in 2021, the focus was on evaluating extracellular vesicles as a potential marker of anaphylaxis. Vesicles were purified from 43 patients at the initial phase of anaphylaxis and under normal conditions. In total, 83 proteins were increased significantly and 16 ones were decreased during the acute phase of anaphylaxis comparing to baseline. Three proteins, namely Cell Division Cycle 42 (CDC42), Ficolin-2 (FCN2), and S100 calcium-binding protein A9 (S100A9), were selected and replicated in other cohort with more samples (25).

Two other studies, conducted *in vitro* using human cells, have shown the changes in the proteome in anaphylaxis (26, 27). The first study demonstrated alterations in the protein expression pattern of human microvascular endothelial cells (ECs) using liquid chromatography coupled to mass spectrometry. In this study, ECs were exposed to sera from individuals experiencing anaphylaxis (EC-anaphylaxis) or from control (EC-control) for a duration of two hours, and the cellular responses were subsequently analyzed. The anaphylactic reactions in patients were triggered by antibiotics or NSAIDs. A total of 1,069 proteins exhibited significant differences between the EC-anaphylaxis and EC-control groups. Notably, 47 proteins showed the most substantial differences, with 38 being increased and 9 decreased. Functional enrichment analysis indicated the involvement of these proteins in blood coagulation, the complement cascade, transport, and extracellular matrix processes (26). In the second study, the aim was to elucidate the mechanism of Tween-80 in the anaphylactoid reaction using LAD2 mast cells. Cells were stimulated with Tween-80 for 30 minutes, and subsequently, label-free LCMS/MS-based proteomics was conducted. Bioinformatics analysis revealed that 313 proteins were upregulated, 111 proteins were downregulated in the Tween-80 group, and an additional 882 proteins were exclusively identified in Tween-80-treated cells. These proteins were implicated in different biological processes, including cellular processes, biological regulation, and metabolic processes, among others. Furthermore, several KEGG pathways involved in the anaphylactoid reactions were identified, such as endocytosis, cell adhesion molecules (CAMs), NF-κB signaling Pathway, and the calcium signaling pathway (27).

*2. Animal model studies:*

The proteome alteration under anaphylaxis condition has been assessed in two studies using an animal model (28, 29). The first study focused on anaphylactoid reaction to Xuesaitong injection (XSTI) that is a natural compound used in the Traditional Chinese Medicine, using a rat model. Samples were collected after 30 min of XSTI injection in two independent experiments. Proteome analysis allowed to identify 13 differentially expressed proteins in the XSTI group. Among these, F13b, Prdx2, Gpx1, Pklr, Ass1, and Asl were up-expressed, while F12, Cndp1, MYLPF, DCD, Kng1, C2, and C6 were down-expressed in the XSTI group. These findings suggest that XSTI triggers an anaphylactoid reaction through direct stimulation, complement activation, and involvement of the kallikrein–kinin pathway (28). In the second study, the aim was to discover protein biomarkers associated with food allergy in mice subjected to ovomucoid exposure (OVM). By Two-Dimensional Difference Gel Electrophoresis, three proteins Hp, SAA2, and PrxII were recognized as being upregulated during OVM-induced anaphylaxis. Although these proteins were detected during anaphylaxis, the authors propose that *i)* they could have been elevated before the challenge, possibly because of repeated gavages during sensitization and *ii)* it is yet uncertain if these proteins are implicated in the mechanism of anaphylaxis or if they are nonspecific proteins associated with bystander injury (29).

Finally, in a study by Hao et al. in 2022, potential anaphylactic effect of saponins were inquired using the RBL-2H3 rat cells. The study investigated differentially expressed proteins caused by several saponins, used in Chinese medicine and possessing distinct anaphylactic capabilities, following a 30-minute incubation with cells. The results indicated that the anaphylactoid reaction of saponins with strong anaphylactic abilities was associated with biological processes responding to organic cyclic compounds and cellular components of the endoplasmic reticulum and Golgi apparatus, which are closely linked to anaphylactoid reactions. Four downregulated proteins were uniquely identified as differentially expressed in the strong anaphylactic groups, suggesting their potential key roles in the anaphylactoid reactions of these cells. These proteins include A0A0G2JWQ0 (Gtf2h1), D3ZL85 (Hccs), D4A5G8 (Pdha1l1), and Q8K3F0 (UniProtKB unreviewed/TrEMBL) (30).

#### 3.1.2 Meta-analysis of proteome data

**Figure 2:**
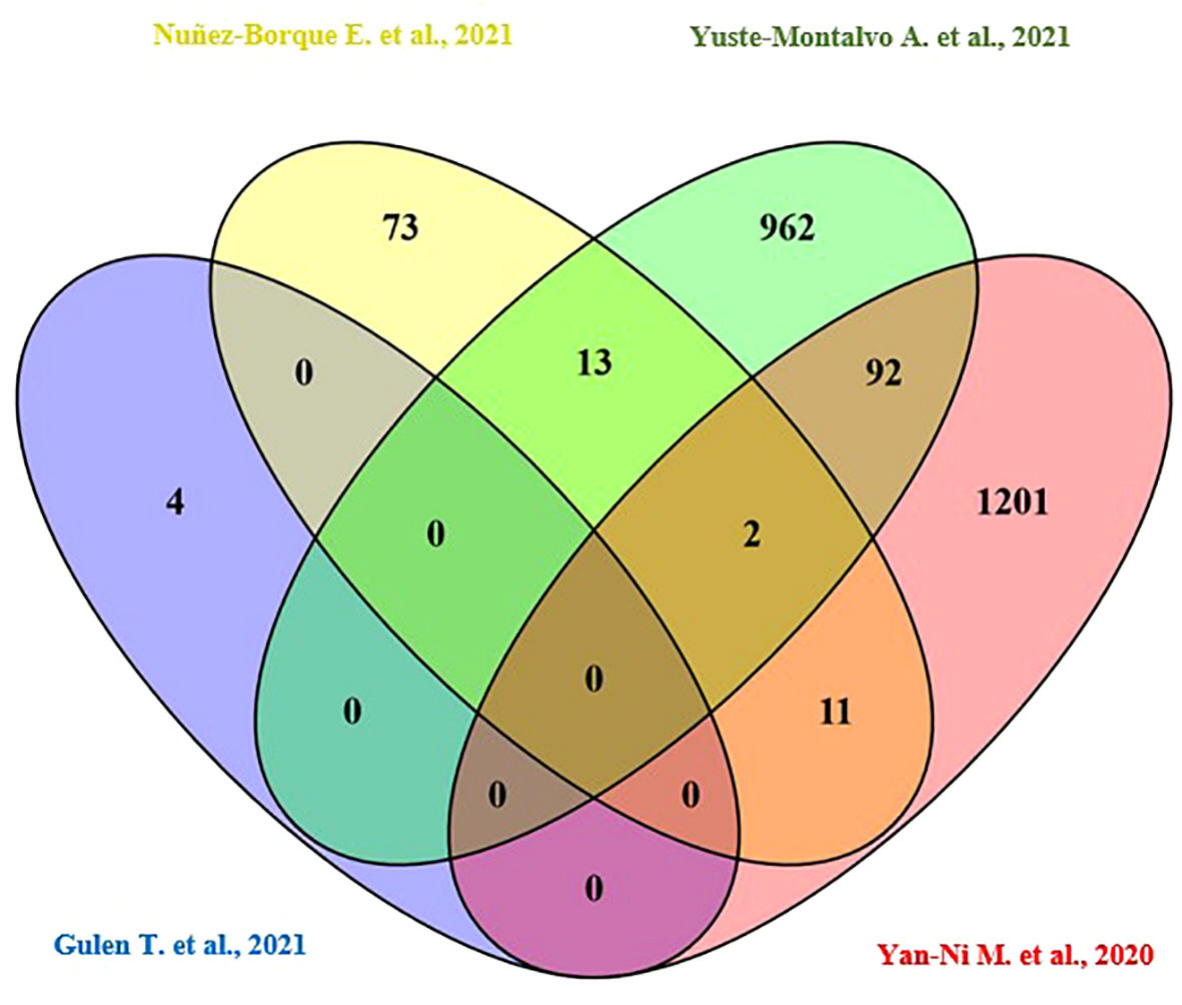
A Venn diagram is used to represent the number of common proteins with differential expression levels, revealing that 118 proteins were found in at least two human studies.

**Figure 3:**
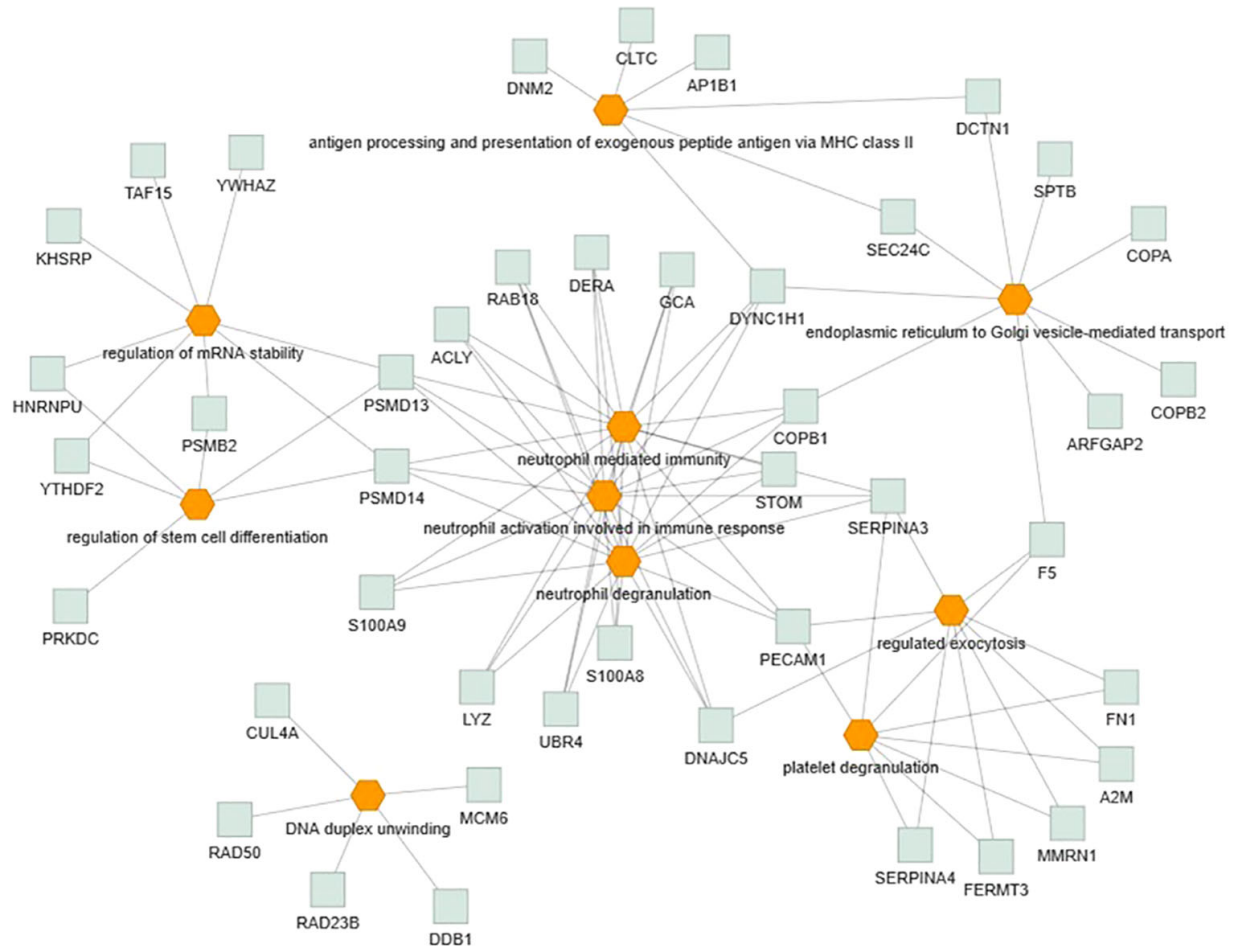
Functional enrichment analysis, using Flame database, of the 118 common proteins identified in this systematic review with an integrated network of the top ten enriched biological processes terms (*P*-value < 0.05). Orange = GO_BP, Grey = overlapping proteins within the GO term.

**Figure 4:**
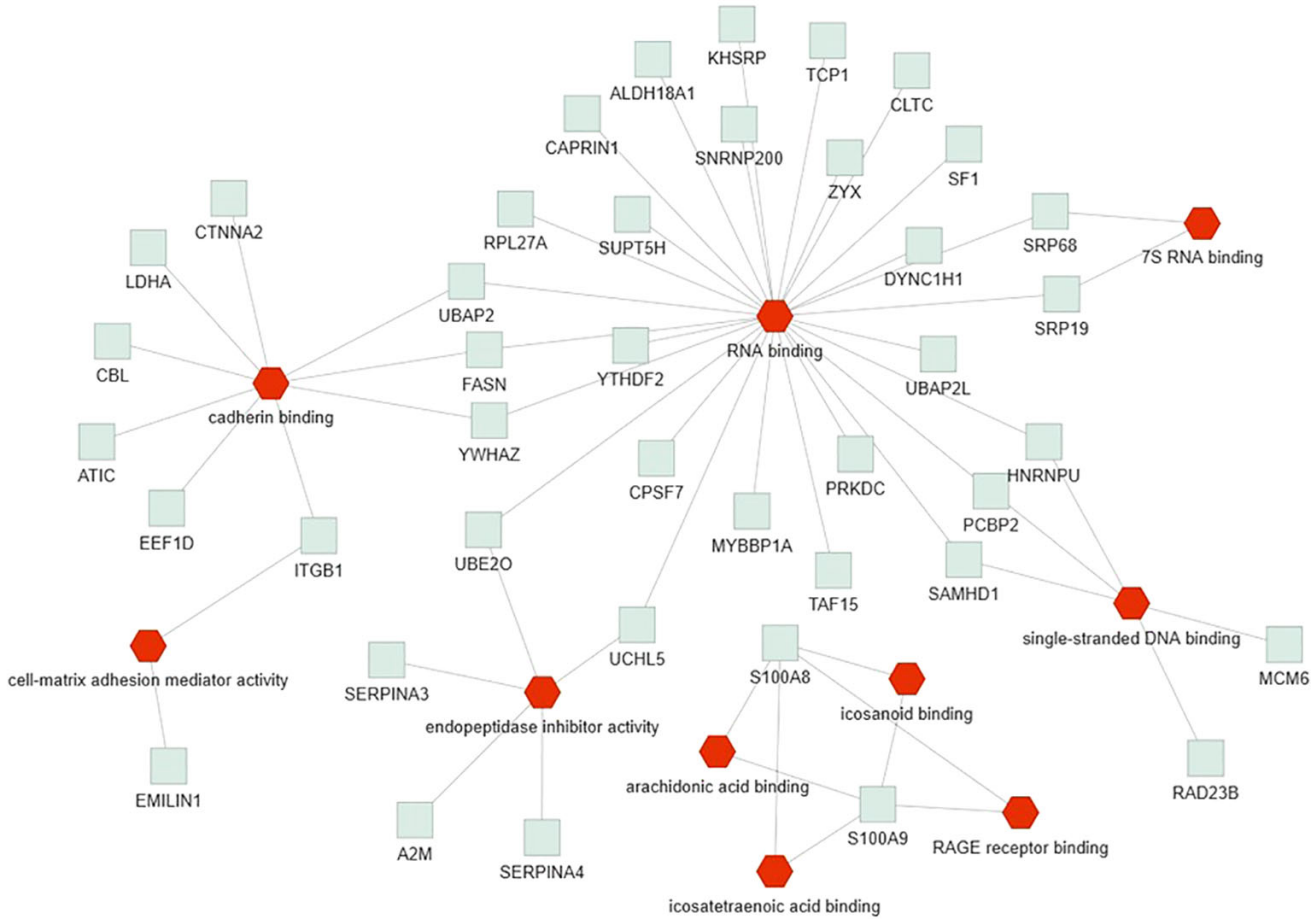
Functional enrichment analysis, using Flame database, of the 118 common proteins identified in this systematic review with an integrated network of the top ten enriched molecular function terms (*P*-value < 0.05). Red = GO_MF, Grey = overlapping proteins within the GO term.

**Figure 5:**
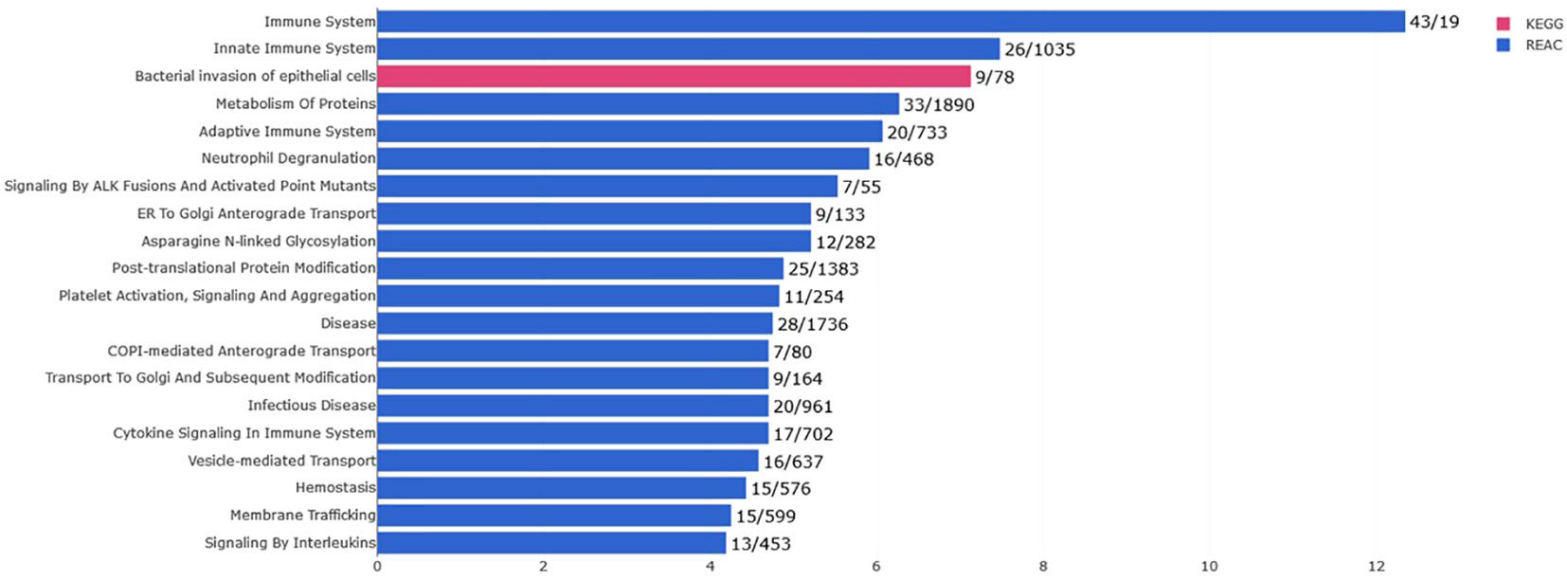
Pathway analysis of the 118 common proteins identified in this systematic review with the top 20 pathways (*P*-value < 0.05, KEGG in pink and REACTOME in blue).

Only the four human studies were included in this analysis (24–27). 118 proteins with different significant levels of expression were found in at least two studies (**Figure 2** and **Table 2**), of which, two proteins were found alerted in three studies, Fibronectin (FINC, Gene name = *FN1*), Integrin beta-1 (ITB1, Gene name = *ITGB1*). Functional Enrichment Analysis using Flame showed 96 significant enriched GO_BP (**Supplementary Table 2**). **Figure 3** shows an integrated network containing the ten top enriched terms connected to their overlapping proteins. It is involved in “GO:0002283~ neutrophil activation involved in immune response, GO:0002446~neutrophil mediated immunity, GO:0043312~neutrophil degranulation, GO:0002576~platelet degranulation”. In the same way, this analysis shows 21 significant enriched GO_MF (**Supplementary Table 2**). **Figure 4** visualizes the connections between the top ten enriched terms and their proteins. It is included “GO:0003723~RNA binding, GO:0045296~cadherin binding, GO:0004866~ endopeptidase inhibitor activity, GO:0003697~single-stranded DNA binding, GO:0050543~ icosatetraenoic acid binding, GO:0050544~arachidonic acid binding”. In addition, pathway analysis identified enrichment for pathways related to innate and adaptive immune system, neutrophil degranulation, and platelet activation (**Figure 5**). **Supplementary Table 2** presents enriched pathways, including the genes mapped to each pathway and their respective significance degrees.

**Table 2:** List of proteins with altered expression levels in anaphylaxis conditions reported in at least two human studies.

Accession number	Protein name	Gene Name	Description	Expression level (26)	Expression level (27)	Expression level (25)
P01023	A2M	A2M	Alpha-2-macroglobulin	NA	↑	↓
P61221	ABCE1	ABCE1	ATP-binding cassette sub-family E member 1	↓	↑	NA
P53396	ACLY	ACLY	ATP-citrate synthase	↓	NA	↑
P43652	AFAM	AFM	Afamin	↑	NA	↓
Q8IZ83	ALDH16A1	ALDH16A1	Aldehyde dehydrogenase family 16 member A1	NA	↑	↑
P54886	P5CS	ALDH18A1	Delta-1-pyrroline-5-carboxylate synthase	↓	↑	NA
Q10567	AP1B1	AP1B1	AP-1 complex subunit beta-1	↓	↑	NA
Q8N6H7	ARFG2	ARFGAP2	ADP-ribosylation factor GTPase-activating protein 2	↓	↑	NA
O43681	ASNA	ASNA1	ATPase ASNA1	↓	↑	NA
P31939	PUR9	ATIC	Bifunctional purine biosynthesis protein PURH	↓	↑	NA
P36542	ATPG	ATP5C1	ATP synthase subunit gamma, mitochondrial	↓	↑	NA
J3KN59	J3KN59	BNIP2	BCL2/adenovirus E1B 19 kDa protein-interacting protein 2	↑	↑	NA
P27708	PYR1	CAD	CAD protein	↓	↑	NA
Q14444	CAPR1	CAPRIN1	Caprin-1	↓	↑	NA
P22681	CBL	CBL	E3 ubiquitin-protein ligase CBL	↓	↑	NA
P60953	CDC42	CDC42	Cell division control protein 42 homolog	↓	NA	↑
A0A087WVQ6	CLTC	CLTC	Clathrin heavy chain	NA	↑	↑
P53621	COPA	COPA	Coatomer subunit alpha	↓	↑	NA
P53618	COPB	COPB1	Coatomer subunit beta	↓	↑	NA
P35606	COPB2	COPB2	Coatomer subunit beta’	↓	↑	NA
Q8N684	CPSF7	CPSF7	Cleavage and polyadenylation specificity factor subunit 7	↓	↑	NA
P26232	CTNA2	CTNNA2	Catenin alpha-2	↓	↑	NA
P17812	PYRG1	CTPS1	CTP synthase 1	↓	↑	NA
Q13619	CUL4A	CUL4A	Cullin-4A	↓	↑	NA
P00167	CYB5	CYB5A	Cytochrome b5	↓	↑	NA
Q14203	DCTN1	DCTN1	Dynactin subunit 1	↓	↑	NA
Q16531	DDB1	DDB1	DNA damage-binding protein 1	↓	↓	NA
Q5TDH0	DDI2	DDI2	Protein DDI1 homolog 2	↑	↑	NA
Q9Y315	DEOC	DERA	Deoxyribose-phosphate aldolase	↑	NA	↑
Q9H3Z4	DNJC5	DNAJC5	DnaJ homolog subfamily C member 5	↑	↑	NA
P50570	DYN2	DNM2	Dynamin-2	↓	↑	NA
Q14204	DYHC1	DYNC1H1	Cytoplasmic dynein 1 heavy chain 1	↓	↑	NA
E9PRY8	E9PRY8	EEF1D	Elongation factor 1-delta OS=Homo sapiens	↓	↑	NA
Q12805	FBLN3	EFEMP1	EGF-containing fibulin-like extracellular matrix protein 1	↑	NA	↑
Q92556	ELMO1	ELMO1	Engulfment and cell motility protein 1	↓	↑	NA
Q9Y6C2	EMIL1	EMILIN1	EMILIN-1	↓	NA	↑
P07814	SYEP	EPRS	Bifunctional glutamate/proline–tRNA ligase	↓	↑	NA
A0A0A0MRJ7	F5	F5	Coagulation factor V	NA	↑	↑
P49327	FAS	FASN	Fatty acid synthase	↓	↑	NA
Q86UX7	FERMT3	FERMT3	Fermitin family homolog 3	NA	↑	↑
Q13045	FLII	FLII	Protein flightless-1 homolog	↓	↑	NA
P02751	FINC	FN1	Fibronectin	↓	↑	↑
P28676	GRAN	GCA	Grancalcin	↑	↑	NA
Q06210	GFPT1	GFPT1	Glutamine–fructose-6-phosphate aminotransferase [isomerizing] 1	↓	↑	NA
Q96IJ6	GMPPA	GMPPA	Mannose-1-phosphate guanyltransferase alpha	↓	↑	NA
P04899	GNAI2	GNAI2	Guanine nucleotide-binding protein G(i) subunit alpha-2	NA	↑	↑
P02042	HBD	HBD	Hemoglobin subunit delta	↑	NA	↑
P05114	HMGN1	HMGN1	Non-histone chromosomal protein HMG-14	↓	↓	NA
Q00839	HNRPU	HNRNPU	Heterogeneous nuclear ribonucleoprotein U	↓	↑	NA
P02790	HEMO	HPX	Hemopexin	↑	NA	↓
Q27J81	INF2	INF2	Inverted formin-2	↓	↑	NA
O95373	IPO7	IPO7	Importin-7	↓	↑	NA
P05556	ITB1	ITGB1	Integrin beta-1	↓	↑	↑
Q92945	FUBP2	KHSRP	Far upstream element-binding protein 2	↓	↑	NA
P08779	KRT16	KRT16	Keratin, type I cytoskeletal 16	NA	↑	↑
P18428	LBP	LBP	Lipopolysaccharide-binding protein	↑	NA	↑
P00338	LDHA	LDHA	L-lactate dehydrogenase A chain	↓	↑	NA
O75427	LRCH4	LRCH4	Leucine-rich repeat and calponin homology domain-containing protein 4	↑	↑	NA
P61626	LYSC	LYZ	Lysozyme C	↑	↓	NA
Q14566	MCM6	MCM6	DNA replication licensing factor MCM6	↓	↑	NA
Q13201	MMRN1	MMRN1	Multimerin-1	↓	NA	↑
Q9H2W6	RM46	MRPL46	39S ribosomal protein L46, mitochondrial	↓	↑	NA
Q9BQG0	MBB1A	MYBBP1A	Myb-binding protein 1A	↓	↑	NA
O00483	NDUA4	NDUFA4	Cytochrome c oxidase subunit NDUFA4	↓	↑	NA
Q9BZD4	NUF2	NUF2	Kinetochore protein Nuf2	↓	↑	NA
Q92621	NU205	NUP205	Nuclear pore complex protein Nup205	↓	↑	NA
Q15366	PCBP2	PCBP2	Poly(rC)-binding protein 2	↓	↑	NA
Q9NR12	PDLI7	PDLIM7	PDZ and LIM domain protein 7	↓	↑	NA
A0A075B738	PECAM1	PECAM1	Platelet endothelial cell adhesion molecule	NA	↑	↑
P52209	6PGD	PGD	6-phosphogluconate dehydrogenase, decarboxylating	↓	↑	NA
Q16512	PKN1	PKN1	Serine/threonine-protein kinase N1	↓	↑	NA
Q8TCU6	PREX1	PREX1	Phosphatidylinositol 3,4,5-trisphosphate-dependent Rac exchanger 1 protein	↓	↑	NA
P10644	KAP0	PRKAR1A	cAMP-dependent protein kinase type I-alpha regulatory subunit	↓	↑	NA
P78527	PRKDC	PRKDC	DNA-dependent protein kinase catalytic subunit	↓	↑	NA
P49721	PSB2	PSMB2	Proteasome subunit beta type-2	↓	↑	NA
Q9UNM6	PSD13	PSMD13	26S proteasome non-ATPase regulatory subunit 13	↓	↑	NA
O00487	PSDE	PSMD14	26S proteasome non-ATPase regulatory subunit 14	↓	↑	NA
Q9NP72	RAB18	RAB18	Ras-related protein Rab-18	↓	↑	NA
P54727	RD23B	RAD23B	UV excision repair protein RAD23 homolog B	↓	↓	NA
Q92878	RAD50	RAD50	DNA repair protein RAD50	↓	↑	NA
P49792	RBP2	RANBP2	E3 SUMO-protein ligase RanBP2	↓	↑	NA
P54136	SYRC	RARS	Arginine–tRNA ligase, cytoplasmic	↓	↑	NA
P46776	RL27A	RPL27A	60S ribosomal protein L27a	↓	↑	NA
P05109	S100A8	S100A8	Protein S100-A8	NA	↓	↑
P06702	S100A9	S100A9	Protein S100-A9	NA	↓	↑
Q9Y3Z3	SAMH1	SAMHD1	Deoxynucleoside triphosphate triphosphohydrolase SAMHD1	↓	↑	NA
P53992	SC24C	SEC24C	Protein transport protein Sec24C	↓	↑	NA
P01011	AACT	SERPINA3	Alpha-1-antichymotrypsin	↑	NA	↓
P29622	KAIN	SERPINA4	Kallistatin	↑	NA	↑
Q15637	SF01	SF1	Splicing factor 1	↓	↑	NA
P29353	SHC1	SHC1	SHC-transforming protein 1	↑	↑	NA
Q96FS4	SIPA1	SIPA1	Signal-induced proliferation-associated protein 1	↓	↑	NA
Q15043	S39AE	SLC39A14	Zinc transporter ZIP14	↑	↑	NA
O75643	U520	SNRNP200	U5 small nuclear ribonucleoprotein 200 kDa helicase	↓	↑	NA
P11277	SPTB1	SPTB	Spectrin beta chain, erythrocytic	↓	NA	↑
P09132	SRP19	SRP19	Signal recognition particle 19 kDa protein	↑	↓	NA
Q9UHB9	SRP68	SRP68	Signal recognition particle subunit SRP68	↓	↑	NA
Q9Y5M8	SRPRB	SRPRB	Signal recognition particle receptor subunit beta	↓	↑	NA
P27105	STOM	STOM	Erythrocyte band 7 integral membrane protein	NA	↑	↑
Q96I99	SUCB2	SUCLG2	Succinate–CoA ligase [GDP-forming] subunit beta, mitochondrial	↓	↑	NA
O00267	SPT5H	SUPT5H	Transcription elongation factor SPT5 OS=Homo sapiens	↓	↑	NA
Q92804	RBP56	TAF15	TATA-binding protein-associated factor 2N	↓	↑	NA
P26639	SYTC	TARS	Threonine–tRNA ligase, cytoplasmic	↓	↑	NA
P17987	TCPA	TCP1	T-complex protein 1 subunit alpha	↓	↑	NA
Q5JTV8	TOIP1	TOR1AIP1	Torsin-1A-interacting protein 1	↓	↑	NA
Q9C037	TRIM4	TRIM4	E3 ubiquitin-protein ligase TRIM4	↓	↑	NA
P40222	TXLNA	TXLNA	Alpha-taxilin	↓	↑	NA
A0AVT1	UBA6	UBA6	Ubiquitin-like modifier-activating enzyme 6	↓	↑	NA
Q5T6F2	UBAP2	UBAP2	Ubiquitin-associated protein 2	↓	↑	NA
Q14157	UBP2L	UBAP2L	Ubiquitin-associated protein 2-like	↓	↑	NA
Q9C0C9	UBE2O	UBE2O	(E3-independent) E2 ubiquitin-conjugating enzyme	↓	↑	NA
Q5T4S7	UBR4	UBR4	E3 ubiquitin-protein ligase UBR4	↓	↑	NA
Q9Y5K5	UCHL5	UCHL5	Ubiquitin carboxyl-terminal hydrolase isozyme L5	↑	↑	NA
Q9UBQ0	VPS29	VPS29	Vacuolar protein sorting-associated protein 29	↓	↑	NA
Q96QK1	VPS35	VPS35	Vacuolar protein sorting-associated protein 35	↓	↑	NA
Q9Y5A9	YTHD2	YTHDF2	YTH domain-containing family protein 2	↓	↑	NA
P63104	1433Z	YWHAZ	14-3-3 protein zeta/delta	↓	↓	NA
Q15942	ZYX	ZYX	Zyxin	↓	↑	NA

↑, Increased level, ↓, Decreased level, NA: Not Applicable.

### 3.2 Anaphylaxis and metabolome

#### 3.2.1 Literature search findings

The first search approach “anaphylaxis and metabolome“ yielded 39 citations, and after excluding 34 studies, five ones remained eligible for inclusion in this review (28, 31–34). (**Figure 1** and **Supplementary Table 3**) Among them, one assessed the metabolome in human studies (31) and four in animal models (28, 32–34) (**Table 3**).

**Table 3:** Studies that evaluated metabolome alteration in anaphylactic condition.

First author Year		Geographical region	Sample type	Phenotype	Cases (n°)	Controls (n°)	Primary aim	Proteomics study method	Main finding	Ref.
*in vivo* human studies										
**Perales-Chorda, C.** **2021**	Spain		Patients’ serums	Food, drug or idiopathic	18 patients: T1 (onset of the reaction), T2 (after 2/4h)	18 same patients: after 2/3 months (T0)	To study metabolic changes in patients with anaphylactic reactions, considering the trigger and the severity.	Liquid chromatography coupled to mass spectrometry and proton nuclear magnetic resonance spectroscopy.	Comparisons between the onset of the anaphylactic reactions and after 2-4 h revealed distinct metabolic changes in drugs, food and food + drug. Alterations were also observed among metabolites, including glucose, lipids, cortisol, betaine, and oleamide when examined metabolic differences between moderate vs. severe anaphylaxis at the acute phase and the basal state.	(31)
*in vivo* animal model studies										
**Gao, X.** **2021**		China	Male Sprague-Dawley rats	Qingkailing injection (QKLI)	n=7 rats	n=7 rats	To establish an integrative metabolomic approach to unravel the biochemical mechanism of anaphylaxis induced by QKLI.	Ultra-performance liquid chromatography coupled with quadrupole time-of-flight mass spectrometry analysis.	During the early, mid, and late stages of anaphylaxis 58, 59, and 41 metabolites were respectively identified. Notably, 24 of them exhibited consistent trends across all three stages. These findings suggest a close association between the pathogenesis of anaphylaxis induced by QKLI and arachidonic acid metabolism.	(32)
**Xu, Y.** **2017**		China	Male BN rats	Compound 4880 or ovalbumin	n=16 rats 8 + 8 C4880, OVA	n=8 rats	To study the mechanism of the anaphylactoid effect and offer a reference for potential future diagnostics.	Ultra-performance liquid chromatography coupled with quadrupole time-of-flight mass spectrometry analysis.	A total of thirty metabolites were identified as significantly distinguishing anaphylactoid reaction groups. Notably, arachidonic acid, LTA4, LTB4, 5-HPETE, and 5-OxoETE.	(33)
**Yubin, X.** **2015**		China	Male BN rats	Xuesaitong injection (XSTI)	n=8 and n=8 (biological replicate)	n=8 and n=8 (biological replicate)	To explore the anaphylactoid response triggered by herbal medicine.	Ultra-performance liquid chromatography coupled with quadrupole time-of-flight mass spectrometry analysis.	16 increased metabolites and 12 decreased ones were identified in the XSTI group in comparison with the control.	(28)
**Xia, H.** **2012**		China	Guinea pigs	Ovalbumin or cattle albumin	n=24 12 + 12 ovalbumin cattle albumin	n=12	To perform serum metabolic profiling in anaphylaxis animal models and identify potential biomarkers associated with anaphylaxis.	Gas chromatography-mass spectrometry.	22 metabolites with significant differences between the cattle albumin group and the control group, and 19 metabolites between the ovalbumin group and the control group were found.	(34)

*1. Human studies*

Only one study investigated changes in the metabolome of patients with anaphylaxis, focused on reactions triggered by food or drugs (31). Serum samples were collected from adult patients at three distinct time intervals: T1 at the onset of the reaction, T2 approximately 2 to 4 hours later, and T0 after a 2 to 3-month period serving as baseline state. Metabolic alterations were measured by employing ultra-performance liquid chromatography coupled with mass spectrometry (UPLC-MS) and proton nuclear magnetic resonance spectroscopy (H-NMR). The metabolomics profiles tracked the temporal evolution of anaphylaxis by comparing metabolites at the different time points and scrutinized the severity of the response at time points. Only food-induced anaphylaxis exhibited discernible metabolic alterations at T1 *vs*. T2, with a remarkable stratification of patients in principal component analysis. A total of 73 metabolites were identified as significantly altered, primarily involved in phospholipid-related pathways. A notable increase in acetate, phenylalanine, lysine, creatine, and glutamine at T1 was also reported.

Regarding the association with severity, this study identified more pronounced metabolic alterations in patients with moderate reactions at T1 compared to T2. Indeed, individuals with moderate reactions displayed heightened levels of choline, lactate, acetate, arginine, glutamine, isoleucine, leucine, valine, phenylalanine, proline, and creatinine and reduced levels of phosphatidylcholines (PCs) and phosphatidylethanolamines (PEs) at T1. Conversely, an increase in PEs and PCs metabolites was observed among patients with severe reactions at T1.

In addition, the study inquired the differences in metabolite profiles between two severity levels at the basal state T0 and at T1. Comparing the severe vs moderate groups showed an alteration of seven metabolites at T1 and ten metabolites at T0. Regardless of the time point, lipids and glucose levels were elevated in the severe group; Cortisol was increased at T1 and decreased at the baseline T0 (31).

*2. Animal model studies*

Four articles explored changes in metabolic profiles in animal models of anaphylaxis (28, 32–34). The first study evaluated the metabolome profile in anaphylaxis induced by Qingkailing injection (QKLI-IA) in a rat model (32). QKLI-IA is a traditional Chinese medicine employed for upper respiratory tract inflammation treatment. Metabolomics analysis was performed at early (10 minutes), intermediate (30 minutes), and late (120 minutes) phase of anaphylactic reaction using ultra-performance liquid chromatography coupled to quadrupole time-of-flight mass spectrometry in both positive and negative ion modes. The results revealed 58 potentially altered metabolites during the initial stages of the reaction, with 14 of them being as top-ranked candidates by statistical analysis, including principal component analysis and receiver operator characteristic curves. These include creatine, 15-Hydroxyeicosatetraenoic acid (15-HETE), LysoPC (18:1), Sphingosine 1-phosphate (S1P), 5-Hydroxyeicosatetraenoic acid (5-HETE), 12-Hydroxyeicosatetraenoic acid (12-HETE), arachidonic acid, palmitic acid, LysoPC (20:4), PC (16:0/22:6), PC (18:2/16:0), PC (18:0/22:6), PC (18:0/20:3) and diglyceride (DG) (20:4/15:0/0:0) with sensitivity and specificity exceeding 0.85 for each. These metabolites play roles in arachidonic acid, glycerophospholipid, fatty acid, unsaturated fatty acids, and amino acid pathways. In the intermediate phase, 59 potential biomarkers were identified. Notably, 13 of them exhibited significant distinctions between allergic and control rats, including tryptophan, sphinganine, PC (18:3/19:1), PC (22:6/18:0), tyramine, LysoPC (18:4), phosphocholine, SM (d18:1/24:1), and DG (18:0/20:4), ranking as top candidates with sensitivity and specificity exceeding 0.80. These metabolites were associated with glycerophospholipid metabolism, sphingolipid, tryptophan, tyrosine, alpha-linolenic acid, and linoleic acid pathways. In the late phase of QKLI-IA, 41 potential biomarkers were identified, linked to glycerophospholipids, linoleic acid, alpha-linolenic acid, and arachidonic acid pathways. A comparative analysis across the three stages uncovered 24 metabolites associated with arachidonic acid metabolism (32). Xu et al. evaluated the responses to ovalbumin and Compound 4880 in rats after 30 minutes of injection. The study reported changes in concentrations of Arachidonic acid, leukotriene A4 (LTA4), leukotriene B4 (LTB4), 5-HPETE, and 5-OxoETE. Additionally, significant concentration changes were observed for adenosine, histamine, N-acetylhistamine, N(α)-γ-glutamylhistamine, malate, and xanthine (33).

In another study, the same authors sought to elucidate anaphylactoid reactions triggered by the injection of an herbal medicine, Xuesaitong injection (XSTI). They identified 28 metabolites with increased levels of histamine, adenosine, adenine, uric acid, glutathione, leukotriene B4 (LTB4), citric acid, hippuric acid, N-acetylhistamine, N(α)-γ-l-glutamylhistamine, valine, 3-methylhistidine, isocitrate, oxalosuccinate, fumarate, and malate, and decreased levels of alanine, arginine, tryptophan, serine, aspartic acid, arachidonic acid, α-ketoglutaric acid, octadecanoic acid, LTA4, 5-hydroperoxyeicosatetraenoic acid (5-HPETE), indoleacrylic acid, and 1-pyrroline-2-carboxylic acid in the XSTI group. These metabolites are implicated in histidine, purine, and energy metabolisms (28). Metabolites analyzed by gas chromatography-mass spectrometry were evaluated in food anaphylaxis using guinea pigs challenged for one hour. The study included three groups: control, ovalbumin-sensitized, and cattle albumin-sensitized. A total of 22 metabolites showed significant differences in the cattle albumin, while 19 differed in the ovalbumin group compared to control. Nine variables were consistently altered in both treated groups. Notably, six were provisionally identified. Glucose, fumaric acid, L-arabitol were decreased, and n-pentadecanoic acid, (R)-3-hydroxybutyric acid, and myo-inositol were increased in the treated groups. These findings suggest a potential link between anaphylaxis, energy metabolism, and signaling processes (34).

#### 3.2.2 Meta-analysis of metabolome data

We analyzed variations in metabolites in the five identified studies while considering the heterogeneity of the model type, trigger (food or drug), and different sampling time points, resulting in the identification of 13 common metabolites (**Table 4**). Specifically, we observed consistent downregulation across studies by Perales-Chorda C et al. and Gao X. et al. of a metabolite, LysoPC(18:0) lysophosphatidylcholines. Furthermore, we also found upregulation in both studies by Xu Y et al. for metabolites implicated in the Krebs cycle, including isocitrate, oxalosuccinate, fumarate, and malate.

**Table 4:** List of metabolites with altered levels in anaphylaxis conditions reported in at least two studies.

Metabolite Name	Metabolite level (31)	Metabolite level (32)	Metabolite level (33)	Metabolite level (28)	Metabolite level (34)
Arachidonic acid	NA	↑ (#; ##; ###)	NA	↓	NA
Tryptophan	NA	↓ (##)	NA	↓	NA
Isocitrate	NA	NA	↑	↑	NA
Oxalosuccinate	NA	NA	↑	↑	NA
Fumarate	NA	NA	↑	↑	NA
Malate	NA	NA	↑	↑	NA
Creatine	↑ (a, b)	↓ (#; ##; ###)	NA	NA	NA
LysoPC(18:0)	↓ (b)	↓ (#; ##)	NA	NA	NA
Valine	↑ (b)	NA	NA	↑	NA
Arginine	↑ (b)	NA	NA	↓	NA
Serine	↑ (b)	NA	NA	↓	NA
Glucose	↑ (c)	NA	NA	NA	↓

a: Food anaphylaxis between T1 and T2 time points.

b: Moderate anaphylaxis between T1 and T2 time points.

c: Severe vs Moderate anaphylaxis at T1 time point.

# 10 min post-anaphylactic reaction initiation.

## 30 min post-anaphylactic reaction initiation.

### 120 min post-anaphylactic reaction initiation. ↑, Increased level ; ↓, Decreased level ; NA, Not Applicable.

## 4 Discussion

This systematic review identified solely twelve eligible publications, encompassing seven studies assessing proteome analysis in anaphylaxis context, as well as five publications that investigated the association between anaphylaxis and the metabolome. In a study conducted by Yuste-Montalvo A. et al. in 2021, protein patterns in endothelial cell model were identified after exposure to serum from patients in during the acute phase of an anaphylactic reaction (26). The study sheds light on the significance of the coagulation, fibrinolytic and complement systems in human anaphylaxis. Specifically, the activation of the coagulation and fibrinolytic systems frequently occurs simultaneously with the activation of the complement system, serving as a trigger of anaphylaxis (35). Another study suggests that the allergen, like Tween-80, could potentially trigger mediator release after endocytosis (27). This internalization process activates degranulation through the phosphoinositide phospholipase Cγ (PLCγ)/protein kinase C (PKC) pathways, inducing calcium influx and inflammatory mediators release via the NF-κB pathway (27). Indeed, endocytosis is recognized as an important mechanism for the internalization of allergic agents or stimulants by mast cells. Receptor-mediated endocytosis in mast cells involves various receptors, such as FcϵRI (36), Mas-related G protein-coupled receptors X2 (MRGPRX2) (37), and epidermal growth factor receptor (EGFR) (38). The activation of mast cells leads to: *i)* increases the expression of cell adhesion molecules, thereby facilitating the communication and recruitment of other cells during inflammation and hypersensitivity (39), *ii)* the release of both preformed and newly synthesized inflammatory mediators. Nuclear translocation, involving the Mitogen activated protein kinase (MAPK) pathway and the nuclear factor kappa light chain enhancer of activated B cells (NF-κB) pathway, plays a crucial role in the synthesis of new inflammatory mediators (40, 41).

Despite variations in research protocols, allergen types, study models, and bioinformatic analyses across the human studies included in this review, we identified 118 proteins present in at least two independent studies. The biological processes involved in this protein set are focused on immune response and neutrophil function. Data from diverse studies suggests that anaphylactic reactions cannot be exclusively attributed to mast cells and basophils as the principal cellular contributors. Neutrophils and macrophages activation has also been associated with severe allergic reactions, especially those induced by food allergens (42–44). Do et al. published a transcriptomic and epigenomic study in which they found four groups of CpGs and genes, associated with the activities of macrophage and neutrophil in immunity, in relation with the severity of reactions to peanut (43). Indeed, the activation of neutrophils can be initiated by the recognition of food antigens through the binding of IgG to Fc gamma receptors (45), resulting in the release of PAF. This mechanism implicates neutrophils in the pathogenesis of anaphylaxis (46, 47). Jönsson F. et al. provide evidence supporting the role of IgG neutrophil PAF pathway in anaphylaxis provoked by neuromuscular-blocking agents in humans. This pathway may exacerbate anaphylaxis in conjunction with the IgE pathway or potentially underlie anaphylaxis in situations where specific IgE is not present (48). When activated, neutrophils might be capable of triggering anaphylactic symptoms due to their rapid release of potent lipid and protein mediators. Additionally, they can expel their nuclear and mitochondrial DNA, in the form of Neutrophil Extracellular Traps (NETs) through a process called NETosis (49, 50). In addition, our analysis revealed that among the most significant molecular function enriched within the common proteins are icosatetraenoic acid binding and arachidonic acid (AA) binding. Furthermore, AA was significantly altered in two studies (28, 32) after 10, 30 and 120 min post-anaphylactic reaction initiation to QKLI-IA (32). AA acts as the initial substance for producing eicosanoids, powerful inflammation mediators associated with the development of diverse diseases (51, 52). Eicosanoids are primarily produced through the enzymatic actions of cyclooxygenase (also known as prostaglandin endoperoxide synthase), responsible for generating prostaglandins and thromboxane, as well as 5-lipoxygenase, leading to the synthesis of leukotrienes that can contribute to the symptoms of anaphylaxis, such as bronchoconstriction and augmented vascular permeability (52, 53). In the context of QKLI-IA drug, the dynamics of metabolic changes across different stages were illuminated, with specific biomarkers exhibiting high predictive ability. These markers also reveal insights into glycerophospholipid, sphingolipid, and tryptophan metabolism pathways (32). Altered levels of tryptophan were also found in another study in anaphylactic reactions to the XSTI drug (28). Tryptophan, an indispensable aromatic amino acid, undergoes metabolism via three primary pathways: the direct conversion by the gut microbiota, the kynurenine pathway in various immune and epithelial cells, and the transformation into serotonin (54). It is important to note that diagnosing anaphylaxis relies on clinical symptoms since there are no definitive biomarkers available. Biomarkers like serum tryptase and histamine may support the diagnosis, but they are not universally altered in all cases and have limitations in sensitivity and specificity. Despite its potential, metabolomics remains underutilized in human anaphylaxis research due to the unpredictability of anaphylactic events and ethical constraints in controlled settings.

Collectively, our systematic review enriches our understanding of anaphylactic reactions, reveals potential biomarkers, and underscores the intricate interplay between clinical manifestations and underlying metabolic processes, paving the way for improved diagnosis, prevention, and management of allergic reactions. Nevertheless, it is important to acknowledge several limitations in this review and analysis. Firstly, the number of published studies available for analysis was relatively small. Secondly, the studies exhibit distinct designs concerning sample types (e.g., patient serum, different cell cultures), allergen triggers (e.g., food, drug antigens), and bio-informatics analyses (e.g., different tools, settings, thresholds). Thirdly, a majority of these studies used small sample sizes and their results are not replicated. In future studies, systems biology network models that can integrate multiple types of omics data could guide the interpretation of proteomic and metabolomic changes associated with anaphylaxis in their metabolic context. For example, Yuste-Montalvo et al. constructed a network of protein-protein interactions from proteomics data of endothelial cells in anaphylaxis patients and controls (26). This functional enrichment analysis identified blood coagulation and the complement cascade as the main altered pathways in anaphylaxis (26).

## 5 Conclusion

Anaphylaxis is a complex and multifaceted allergic response that involves various mediators and pathways, and research in this field continues to uncover new insights. This systematic review emphasizes alterations in the proteome and metabolome under anaphylactic conditions. Several pathogenic pathways have been highlighted, including neutrophil activation, platelet degranulation, and arachidonic acid binding. However, these findings, derived from a limited number of publications, necessitate confirmation in larger human studies and could contribute to the development of new biomarkers for anaphylaxis.

## Data availability statement

The original contributions presented in the study are included in the article/**Supplementary Material**. Further inquiries can be directed to the corresponding authors.

## Author contributions

AG: Conceptualization, Formal analysis, Methodology, Writing – original draft. AH: Writing – review & editing, Supervision. R-MG-R: Writing – review & editing. JLG: Conceptualization, Funding acquisition, Supervision, Validation, Visualization, Writing – review & editing. RS: Conceptualization, Formal analysis, Methodology, Supervision, Validation, Writing – review & editing.

## Supplementary material

Supplementary Table 1 Excluded proteomics studies.

Supplementary Table 2 Functional enrichment and pathway analyses using Flame database: gene ontology biological process (GO-BP), gene ontology molecular function (GO-MF) and pathways (KEGG and REACTOME) terms with a *P*-value < 0.05.

Supplementary Table 3 Excluded metabolomics studies.
