# Supplementary material for: A systematic review and meta-analysis of proteomic and metabolomic alterations in anaphylaxis reactions
Source: Front Immunol. 2024 Feb 7;15:1328212. doi: 10.3389/fimmu.2024.1328212 (PMC10879545; doi:10.3389/fimmu.2024.1328212)
Supplement: Supplementary Table 1 — Excluded proteomics studies. [file Table_1.docx]

**Supplementary Table S1.**

Details of the proteomics studies excluded from the systematic review.

| First author,  Year | Exclusion ground | Reference |
| --- | --- | --- |
| Côrte-Real, B.F.  2023 | No anaphylaxis  No proteome | (1) |
| Packi, K.  2023 | No anaphylaxis | (2) |
| Xu, L.  2022 | No anaphylaxis | (3) |
| Rujitharanawong, C.  2022 | No proteome | (4) |
| Hadadianpour, A.  2022 | No anaphylaxis  No proteome | (5) |
| Radzikowska, U.  2022 | Review paper | (6) |
| Nejabat, S.  2022 | No anaphylaxis  No proteome | (7) |
| Kiweler, N.  2022 | No anaphylaxis  No proteome | (8) |
| Gabler, A.M.  2022 | No proteome | (9) |
| Graham, R.L.J.  2022 | Only mastocytosis | (10) |
| Jeong, K.Y.  2022 | No anaphylaxis  No proteome | (11) |
| Fiocchi, A.  2021 | Review paper | (12) |
| Simionescu, A.A.  2021 | No proteome | (13) |
| Zuberbier, T.  2022 | No anaphylaxis  No proteome | (14) |
| Nuñez-Borque, E.  2021 | No proteome | (15) |
| Moreno-Indias, I.  2021 | Review paper | (16) |
| Czolk, R.  2021 | Review paper | (17) |
| Jappe, U.  2021 | No anaphylaxis  No proteome | (18) |
| AhYoung, A.P.  2020 | No proteome | (19) |
| Grosch, J.  2020 | No anaphylaxis  No proteome | (20) |
| Mendoza-Porras, O.  2020 | No anaphylaxis  No proteome | (21) |
| Cossarizza, A.  2019 | Review paper | (22) |
| Ashtari, S.  2019 | Review paper | (23) |
| Altenbach, S.B.  2018 | No anaphylaxis  No proteome | (24) |
| Ponce, M.  2019 | No anaphylaxis  No proteome | (25) |
| Srisong, H.  2018 | No anaphylaxis  No proteome | (26) |
| Cardona, E.E.G.  2018 | No anaphylaxis  No proteome | (27) |
| Wanandy, T.  2018 | No anaphylaxis  No proteome | (28) |
| Cho, K.  2018 | No anaphylaxis  No proteome | (29) |
| Somiya, M.  2018 | No anaphylaxis  No proteome | (30) |
| Mateos-Hernández, L.  2017 | No proteome | (31) |
| Wittenberg, M.  2017 | No proteome | (32) |
| Xu, Y.  2017 | No anaphylaxis  No proteome | (33) |
| Brandström, J.  2017 | No anaphylaxis | (34) |
| Srisong, H.  2016 | Review paper | (35) |
| Bateman, D.N.  2015 | No proteome | (36) |
| Vidal, C.  2015 | No proteome | (37) |
| Perez-Riverol, A.  2015 | No anaphylaxis  No proteome | (38) |
| Fekecsová, S.  2015 | No anaphylaxis  No proteome | (39) |
| Leung, P.S.  2014 | Review paper | (40) |
| Sookrung, N.  2014 | No anaphylaxis  No proteome | (41) |
| Nakamura, R.  2013 | No anaphylaxis  No proteome  No English language | (42) |
| Barbarroja-Escudero, J.  2014 | No proteome | (43) |
| Uvackova, L.  2013 | No anaphylaxis  No proteome | (44) |
| Kumar, S.  2013 | No anaphylaxis  No proteome | (45) |
| Blank, S.  2012 | No anaphylaxis  No proteome | (46) |
| Altenbach, S.B.  2011 | No anaphylaxis  No proteome | (47) |
| Yamasaki, A.  2010 | No anaphylaxis  No proteome | (48) |
| Dharajiya, N.  2010 | No proteome | (49) |
| Stevenson, S.E.  2009 | No anaphylaxis  No proteome | (50) |
| Hoffman, D.R.  2008 | Review paper | (51) |
| Ou, K.  2001 | No proteome | (52) |

**References**

1. Côrte-Real BF, Hamad I, Arroyo Hornero R, Geisberger S, Roels J, Van Zeebroeck L, Dyczko A, van Gisbergen MW, Kurniawan H, Wagner A, et al. Sodium perturbs mitochondrial respiration and induces dysfunctional Tregs. *Cell Metab* (2023) 35:299-315.e8. doi: 10.1016/j.cmet.2023.01.009

2. Packi K, Matysiak J, Matuszewska E, Bręborowicz A, Matysiak J. Changes in Serum Protein-Peptide Patterns in Atopic Children Allergic to Plant Storage Proteins. *Int J Mol Sci* (2023) 24:1804. doi: 10.3390/ijms24021804

3. Xu L, Zhang XM, Wen YQ, Zhao JL, Xu TC, Yong L, Lin H, Zhang HW, Li ZX. Comparison of tropomyosin released peptide and epitope mapping after in vitro digestion from fish (Larimichthys crocea), shrimp (Litopenaeus vannamei) and clam (Ruditapes philippinarum) through SWATH-MS based proteomics. *Food Chem* (2023) 403:134314. doi: 10.1016/j.foodchem.2022.134314

4. Rujitharanawong C, Yoodee S, Sueksakit K, Peerapen P, Tuchinda P, Kulthanan K, Thongboonkerd V. Systematic comparisons of various markers for mast cell activation in RBL-2H3 cells. *Cell Tissue Res* (2022) 390:413–428. doi: 10.1007/s00441-022-03687-w

5. Hadadianpour A, Daniel J, Zhang J, Spiller BW, Makaraviciute A, DeWitt ÅM, Walden HS, Hamilton RG, Peebles RS, Nutman TB, et al. Human IgE mAbs identify major antigens of parasitic worm infection. *J Allergy Clin Immunol* (2022) 150:1525–1533. doi: 10.1016/j.jaci.2022.05.022

6. Radzikowska U, Baerenfaller K, Cornejo-Garcia JA, Karaaslan C, Barletta E, Sarac BE, Zhakparov D, Villasenor A, Eguiluz-Gracia I, Mayorga C, et al. Omics technologies in allergy and asthma research: An EAACI position paper. *Allergy* (2022) 77:2888–2908. doi: 10.1111/all.15412

7. Nejabat S, Haghshenas MR, Farjadian S. Allergenome profiling of Vespa orientalis venom by serum IgE in patients with anaphylactic reaction to this hornet sting. *Toxicon* (2022) 214:130–135. doi: 10.1016/j.toxicon.2022.05.039

8. Kiweler N, Delbrouck C, Pozdeev VI, Neises L, Soriano-Baguet L, Eiden K, Xian F, Benzarti M, Haase L, Koncina E, et al. Mitochondria preserve an autarkic one-carbon cycle to confer growth-independent cancer cell migration and metastasis. *Nat Commun* (2022) 13:2699. doi: 10.1038/s41467-022-30363-y

9. Gabler AM, Gebhard J, Norwig M-C, Eberlein B, Biedermann T, Brockow K, Scherf KA. Basophil Activation to Gluten and Non-Gluten Proteins in Wheat-Dependent Exercise-Induced Anaphylaxis. *Front Allergy* (2022) 3:822554. doi: 10.3389/falgy.2022.822554

10. Graham RLJ, McMullen AA, Moore G, Dempsey-Hibbert NC, Myers B, Graham C. SWATH-MS identification of CXCL7, LBP, TGFβ1 and PDGFRβ as novel biomarkers in human systemic mastocytosis. *Sci Rep* (2022) 12:5087. doi: 10.1038/s41598-022-08345-3

11. Jeong KY, Lee JS, Yuk JE, Song H, Lee HJ, Kim KJ, Kim BJ, Lim K-J, Park KH, Lee J-H, et al. Allergenic characterization of Bomb m 4, a 30-kDa Bombyx mori lipoprotein 6 from silkworm pupa. *Clin Exp Allergy* (2022) 52:888–897. doi: 10.1111/cea.14095

12. Fiocchi A, Risso D, DunnGalvin A, González Díaz SN, Monaci L, Fierro V, Ansotegui IJ. Food labeling issues for severe food allergic patients. *World Allergy Organ J* (2021) 14:100598. doi: 10.1016/j.waojou.2021.100598

13. Simionescu AA, Stanescu AMA, Popescu F-D. State-of-the-Art on Biomarkers for Anaphylaxis in Obstetrics. *Life (Basel)* (2021) 11:870. doi: 10.3390/life11090870

14. Zuberbier T, Abdul Latiff AH, Abuzakouk M, Aquilina S, Asero R, Baker D, Ballmer-Weber B, Bangert C, Ben-Shoshan M, Bernstein JA, et al. The international EAACI/GA^2^LEN/EuroGuiDerm/APAAACI guideline for the definition, classification, diagnosis, and management of urticaria. *Allergy* (2022) 77:734–766. doi: 10.1111/all.15090

15. Nuñez-Borque E, Fernandez-Bravo S, Rodriguez Del Rio P, Alwashali EM, Lopez-Dominguez D, Gutierrez-Blazquez MD, Laguna JJ, Tome-Amat J, Gallego-Delgado J, Gomez-Lopez A, et al. Increased miR-21-3p and miR-487b-3p serum levels during anaphylactic reaction in food allergic children. *Pediatr Allergy Immunol* (2021) 32:1296–1306. doi: 10.1111/pai.13518

16. Moreno-Indias I, Lahti L, Nedyalkova M, Elbere I, Roshchupkin G, Adilovic M, Aydemir O, Bakir-Gungor B, Santa Pau EC, D’Elia D, et al. Statistical and Machine Learning Techniques in Human Microbiome Studies: Contemporary Challenges and Solutions. *Front Microbiol* (2021) 12:635781. doi: 10.3389/fmicb.2021.635781

17. Czolk R, Klueber J, Sørensen M, Wilmes P, Codreanu-Morel F, Skov PS, Hilger C, Bindslev-Jensen C, Ollert M, Kuehn A. IgE-Mediated Peanut Allergy: Current and Novel Predictive Biomarkers for Clinical Phenotypes Using Multi-Omics Approaches. *Front Immunol* (2020) 11:594350. doi: 10.3389/fimmu.2020.594350

18. Jappe U, Karstedt A, Warneke D, Hellmig S, Böttger M, Riffelmann FW, Treudler R, Lange L, Abraham S, Dölle-Bierke S, et al. Identification and Purification of Novel Low-Molecular-Weight Lupine Allergens as Components for Personalized Diagnostics. *Nutrients* (2021) 13:409. doi: 10.3390/nu13020409

19. AhYoung AP, Eckard SC, Gogineni A, Xi H, Lin SJ, Gerhardy S, Cox C, Phung QT, Hackney JA, Katakam AK, et al. Neutrophil serine protease 4 is required for mast cell-dependent vascular leakage. *Commun Biol* (2020) 3:687. doi: 10.1038/s42003-020-01407-0

20. Grosch J, Hilger C, Bilò MB, Kler S, Schiener M, Dittmar G, Bernardin F, Lesur A, Ollert M, Schmidt-Weber CB, et al. Shedding Light on the Venom Proteomes of the Allergy-Relevant Hymenoptera Polistes dominula (European Paper Wasp) and Vespula spp. (Yellow Jacket). *Toxins (Basel)* (2020) 12:323. doi: 10.3390/toxins12050323

21. Mendoza-Porras O, Kamath S, Harris JO, Colgrave ML, Huerlimann R, Lopata AL, Wade NM. Resolving hemocyanin isoform complexity in haemolymph of black tiger shrimp Penaeus monodon - implications in aquaculture, medicine and food safety. *J Proteomics* (2020) 218:103689. doi: 10.1016/j.jprot.2020.103689

22. Cossarizza A, Chang H-D, Radbruch A, Acs A, Adam D, Adam-Klages S, Agace WW, Aghaeepour N, Akdis M, Allez M, et al. Guidelines for the use of flow cytometry and cell sorting in immunological studies (second edition). *Eur J Immunol* (2019) 49:1457–1973. doi: 10.1002/eji.201970107

23. Ashtari S, Pourhoseingholi MA, Rostami K, Aghdaei HA, Rostami-Nejad M, Busani L, Tavirani MR, Zali MR. Prevalence of gluten-related disorders in Asia-Pacific region: a systematic review. *J Gastrointestin Liver Dis* (2019) 28:95–105. doi: 10.15403/jgld.2014.1121.281.sys

24. Altenbach SB, Chang H-C, Simon-Buss A, Jang Y-R, Denery-Papini S, Pineau F, Gu YQ, Huo N, Lim S-H, Kang C-S, et al. Towards reducing the immunogenic potential of wheat flour: omega gliadins encoded by the D genome of hexaploid wheat may also harbor epitopes for the serious food allergy WDEIA. *BMC Plant Biol* (2018) 18:291. doi: 10.1186/s12870-018-1506-z

25. Ponce M, Schroeder F, Bannert C, Schmidthaler K, Hansen CS, Lindholm Bøgh K, Soldo R, Tsui SK-W, Weinhäusel A, Szépfalusi Z, et al. Preventive sublingual immunotherapy with House Dust Mite extract modulates epitope diversity in pre-school children. *Allergy* (2019) 74:780–787. doi: 10.1111/all.13658

26. Srisong H, Sukprasert S, Klaynongsruang S, Daduang J, Daduang S. Identification, expression and characterization of the recombinant Sol g 4.1 protein from the venom of the tropical fire ant Solenopsis geminata. *J Venom Anim Toxins Incl Trop Dis* (2018) 24:23. doi: 10.1186/s40409-018-0159-6

27. Cardona EEG, Heathcote K, Teran LM, Righetti PG, Boschetti E, D’Amato A. Novel low-abundance allergens from mango via combinatorial peptide libraries treatment: A proteomics study. *Food Chem* (2018) 269:652–660. doi: 10.1016/j.foodchem.2018.06.113

28. Wanandy T, Wilson R, Gell D, Rose HE, Gueven N, Davies NW, Brown SGA, Wiese MD. Towards complete identification of allergens in Jack Jumper (Myrmecia pilosula) ant venom and their clinical relevance: An immunoproteomic approach. *Clin Exp Allergy* (2018) 48:1222–1234. doi: 10.1111/cea.13224

29. Cho K, Beom H-R, Jang Y-R, Altenbach SB, Vensel WH, Simon-Buss A, Lim S-H, Kim MG, Lee J-Y. Proteomic Profiling and Epitope Analysis of the Complex α-, γ-, and ω-Gliadin Families in a Commercial Bread Wheat. *Front Plant Sci* (2018) 9:818. doi: 10.3389/fpls.2018.00818

30. Somiya M, Yoshioka Y, Ochiya T. Biocompatibility of highly purified bovine milk-derived extracellular vesicles. *J Extracell Vesicles* (2018) 7:1440132. doi: 10.1080/20013078.2018.1440132

31. Mateos-Hernández L, Villar M, Moral A, Rodríguez CG, Arias TA, de la Osa V, Brito FF, Fernández de Mera IG, Alberdi P, Ruiz-Fons F, et al. Tick-host conflict: immunoglobulin E antibodies to tick proteins in patients with anaphylaxis to tick bite. *Oncotarget* (2017) 8:20630–20644. doi: 10.18632/oncotarget.15243

32. Wittenberg M, Nassiri M, Francuzik W, Lehmann K, Babina M, Worm M. Serum levels of 9α,11β-PGF2 and apolipoprotein A1 achieve high predictive power as biomarkers of anaphylaxis. *Allergy* (2017) 72:1801–1805. doi: 10.1111/all.13176

33. Xu Y, Guo N, Dou D, Ran X, Liu C. Metabolomics analysis of anaphylactoid reaction reveals its mechanism in a rat model. *Asian Pac J Allergy Immunol* (2017) 35:224–232. doi: 10.12932/AP0845

34. Brandström J, Vetander M, Lilja G, Johansson SGO, Sundqvist A-C, Kalm F, Nilsson C, Nopp A. Individually dosed omalizumab: an effective treatment for severe peanut allergy. *Clin Exp Allergy* (2017) 47:540–550. doi: 10.1111/cea.12862

35. Srisong H, Daduang S, Lopata AL. Current advances in ant venom proteins causing hypersensitivity reactions in the Asia-Pacific region. *Mol Immunol* (2016) 69:24–32. doi: 10.1016/j.molimm.2015.11.003

36. Bateman DN. Changing the Management of Paracetamol Poisoning. *Clin Ther* (2015) 37:2135–2141. doi: 10.1016/j.clinthera.2015.07.012

37. Vidal C, Bartolomé B, Rodríguez V, Armisén M, Linneberg A, González-Quintela A. Sensitization pattern of crustacean-allergic individuals can indicate allergy to molluscs. *Allergy* (2015) 70:1493–1496. doi: 10.1111/all.12693

38. Perez-Riverol A, Justo-Jacomini DL, Zollner R de L, Brochetto-Braga MR. Facing Hymenoptera Venom Allergy: From Natural to Recombinant Allergens. *Toxins (Basel)* (2015) 7:2551–2570. doi: 10.3390/toxins7072551

39. Fekecsová S, Danchenko M, Uvackova L, Skultety L, Hajduch M. Using 7 cm immobilized pH gradient strips to determine levels of clinically relevant proteins in wheat grain extracts. *Front Plant Sci* (2015) 6:433. doi: 10.3389/fpls.2015.00433

40. Leung PSC, Shu S-A, Chang C. The changing geoepidemiology of food allergies. *Clin Rev Allergy Immunol* (2014) 46:169–179. doi: 10.1007/s12016-014-8411-5

41. Sookrung N, Wong-din-Dam S, Tungtrongchitr A, Reamtong O, Indrawattana N, Sakolvaree Y, Visitsunthorn N, Manuyakorn W, Chaicumpa W. Proteome and allergenome of Asian wasp, Vespa affinis, venom and IgE reactivity of the venom components. *J Proteome Res* (2014) 13:1336–1344. doi: 10.1021/pr4009139

42. Nakamura R, Sakai S, Haishima Y, Fukui C, Suzuki T, Nakamura R, Hachisuka A, Adachi R, Teshima R. [Comprehensive analyses of hydrolyzed wheat protein using shotgun proteomics]. *Kokuritsu Iyakuhin Shokuhin Eisei Kenkyusho Hokoku* (2013)50–57.

43. Barbarroja-Escudero J, Antolin-Amerigo D, Sanchez-Gonzalez M-J, Rodriguez-Rodriguez M, Ledesma-Fernandez A, Alvarez-Mon M. Pine nut anaphylaxis: a proteomic study. *Allergol Int* (2014) 63:125–126. doi: 10.2332/allergolint.13-LE-0584

44. Uvackova L, Skultety L, Bekesova S, McClain S, Hajduch M. MS(E) based multiplex protein analysis quantified important allergenic proteins and detected relevant peptides carrying known epitopes in wheat grain extracts. *J Proteome Res* (2013) 12:4862–4869. doi: 10.1021/pr400336f

45. Kumar S, Verma AK, Sharma A, Kumar D, Tripathi A, Chaudhari BP, Das M, Jain SK, Dwivedi PD. Phytohemagglutinins augment red kidney bean (Phaseolus vulgaris L.) induced allergic manifestations. *J Proteomics* (2013) 93:50–64. doi: 10.1016/j.jprot.2013.02.003

46. Blank S, Bantleon FI, McIntyre M, Ollert M, Spillner E. The major royal jelly proteins 8 and 9 (Api m 11) are glycosylated components of Apis mellifera venom with allergenic potential beyond carbohydrate-based reactivity. *Clin Exp Allergy* (2012) 42:976–985. doi: 10.1111/j.1365-2222.2012.03966.x

47. Altenbach SB, Allen PV. Transformation of the US bread wheat “Butte 86” and silencing of omega-5 gliadin genes. *GM Crops* (2011) 2:66–73. doi: 10.4161/gmcr.2.1.15884

48. Yamasaki A, Higaki H, Nakashima K, Yamamoto O, Hein KZ, Takahashi H, Chinuki Y, Morita E. Identification of a major yolk protein as an allergen in sea urchin roe. *Acta Derm Venereol* (2010) 90:235–238. doi: 10.2340/00015555-0783

49. Dharajiya N, Vaidya SV, Murai H, Cardenas V, Kurosky A, Boldogh I, Sur SA. FcgammaRIIb inhibits allergic lung inflammation in a murine model of allergic asthma. *PLoS One* (2010) 5:e9337. doi: 10.1371/journal.pone.0009337

50. Stevenson SE, Chu Y, Ozias-Akins P, Thelen JJ. Validation of gel-free, label-free quantitative proteomics approaches: applications for seed allergen profiling. *J Proteomics* (2009) 72:555–566. doi: 10.1016/j.jprot.2008.11.005

51. Hoffman DR. Structural biology of allergens from stinging and biting insects. *Curr Opin Allergy Clin Immunol* (2008) 8:338–342. doi: 10.1097/ACI.0b013e3283036a7d

52. Ou K, Seow TK, Liang RC, Lee BW, Goh DL, Chua KY, Chung MC. Identification of a serine protease inhibitor homologue in Bird’s Nest by an integrated proteomics approach. *Electrophoresis* (2001) 22:3589–3595. doi: 10.1002/1522-2683(200109)22:16<3589::AID-ELPS3589>3.0.CO;2-J
