# Supplementary material for: A systematic review and meta-analysis of proteomic and metabolomic alterations in anaphylaxis reactions
Source: Front Immunol. 2024 Feb 7;15:1328212. doi: 10.3389/fimmu.2024.1328212 (PMC10879545; doi:10.3389/fimmu.2024.1328212)
Supplement: Supplementary Table 2 — Functional enrichment and pathway analyses using Flame database: gene ontology biological process (GO-BP), gene ontology molecular function (GO-MF) and pathways (KEGG and REACTOME) terms with a P-value < 0.05. [file Table_2.docx]

**Supplementary Table S2.**

Details of the metabolomics studies excluded from the systematic review.

| First author,  Year | Exclusion ground | Reference |
| --- | --- | --- |
| Devonshire, A.  2023 | Review paper | (1) |
| Soriano-Baguet, L.  2023 | No anaphylaxis | (2) |
| Côrte-Real, B.F.  2023 | No anaphylaxis | (3) |
| Del Duca, E.  2022 | No metabolome | (4) |
| Know, A.S.F.  2022 | Study on the treatment effect of herb extract. | (5) |
| Tan, T.J.  2022 | No anaphylaxis | (6) |
| Li, J.  2022 | No anaphylaxis  No metabolome | (7) |
| Obeso, D.  2022 | No anaphylaxis | (8) |
| López-Sanz, C.  2022 | Review paper | (9) |
| Radzikowska, U.  2022 | Review paper | (10) |
| Mansell, T.  2022 | No anaphylaxis | (11) |
| Taketomi, Y.  2022 | Review paper | (12) |
| Suntivich, R.  2022 | No anaphylaxis | (13) |
| De Paepe, E.  2022 | Review paper | (14) |
| Ogulur, I.  2021 | Review paper | (15) |
| Agopian, J.  2021 | No anaphylaxis | (16) |
| Joseph, A.  2020 | No anaphylaxis | (17) |
| Schmitt, C.  2020 | No anaphylaxis | (18) |
| Doña, I.  2019 | Review paper | (19) |
| Beck, S.C.  2019 | Review paper | (20) |
| Murakami, M.  2019 | Review paper | (21) |
| Dinis-Oliveira, R.J.  2018 | Review paper | (22) |
| Khoo, L.W.  2018 | No anaphylaxis | (23) |
| Shimanaka, Y.  2017 | No anaphylaxis | (24) |
| Steinke, J.W.  2016 | No anaphylaxis | (25) |
| Kong, J.  2015 | Not at the anaphylaxis moment | (26) |
| Pettersson, J.  2008 | No anaphylaxis | (27) |

**References**

1. Devonshire A, Gautam Y, Johansson E, Mersha TB. Multi-omics profiling approach in food allergy. *World Allergy Organ J* (2023) 16:100777. doi: 10.1016/j.waojou.2023.100777

2. Soriano-Baguet L, Grusdat M, Kurniawan H, Benzarti M, Binsfeld C, Ewen A, Longworth J, Bonetti L, Guerra L, Franchina DG, et al. Pyruvate dehydrogenase fuels a critical citrate pool that is essential for Th17 cell effector functions. *Cell Rep* (2023) 42:112153. doi: 10.1016/j.celrep.2023.112153

3. Côrte-Real BF, Hamad I, Arroyo Hornero R, Geisberger S, Roels J, Van Zeebroeck L, Dyczko A, van Gisbergen MW, Kurniawan H, Wagner A, et al. Sodium perturbs mitochondrial respiration and induces dysfunctional Tregs. *Cell Metab* (2023) 35:299-315.e8. doi: 10.1016/j.cmet.2023.01.009

4. Del Duca E, Sansone A, Sgrulletti M, Di Nolfo F, Chini L, Ferreri C, Moschese V. Fatty-Acid-Based Membrane Lipidome Profile of Peanut Allergy Patients: An Exploratory Study of a Lifelong Health Condition. *Int J Mol Sci* (2022) 24:120. doi: 10.3390/ijms24010120

5. Kow ASF, Khoo LW, Tan JW, Abas F, Lee M-T, Israf DA, Shaari K, Tham CL. Clinacanthus nutans aqueous leaves extract exerts anti-allergic activity in preclinical anaphylactic models via alternative IgG pathway. *J Ethnopharmacol* (2023) 303:116003. doi: 10.1016/j.jep.2022.116003

6. Tan TJ, Delgado-Dolset MI, Escribese MM, Barber D, Layhadi JA, Shamji MH. Biomarkers of AIT: Models of prediction of efficacy. *Allergol Select* (2022) 6:267–275. doi: 10.5414/ALX02333E

7. Li J, Li L, Liu R, Zhu L, Zhou B, Xiao Y, Hou G, Lin L, Chen X, Peng C. Integrative lipidomic features identify plasma lipid signatures in chronic urticaria. *Front Immunol* (2022) 13:933312. doi: 10.3389/fimmu.2022.933312

8. Obeso D, Contreras N, Dolores-Hernández M, Carrillo T, Barbas C, Escribese MM, Villaseñor A, Barber D. Development of a Novel Targeted Metabolomic LC-QqQ-MS Method in Allergic Inflammation. *Metabolites* (2022) 12:592. doi: 10.3390/metabo12070592

9. López-Sanz C, Jiménez-Saiz R, Esteban V, Delgado-Dolset MI, Perales-Chorda C, Villaseñor A, Barber D, Escribese MM. Mast Cell Desensitization in Allergen Immunotherapy. *Front Allergy* (2022) 3:898494. doi: 10.3389/falgy.2022.898494

10. Radzikowska U, Baerenfaller K, Cornejo-Garcia JA, Karaaslan C, Barletta E, Sarac BE, Zhakparov D, Villasenor A, Eguiluz-Gracia I, Mayorga C, et al. Omics technologies in allergy and asthma research: An EAACI position paper. *Allergy* (2022) 77:2888–2908. doi: 10.1111/all.15412

11. Mansell T, Saffery R, Burugupalli S, Ponsonby A-L, Tang MLK, O’Hely M, Bekkering S, Smith AAT, Rowland R, Ranganathan S, et al. Early life infection and proinflammatory, atherogenic metabolomic and lipidomic profiles in infancy: a population-based cohort study. *Elife* (2022) 11:e75170. doi: 10.7554/eLife.75170

12. Taketomi Y, Miki Y, Murakami M. Old but New: Group IIA Phospholipase A2 as a Modulator of Gut Microbiota. *Metabolites* (2022) 12:352. doi: 10.3390/metabo12040352

13. Suntivich R, Songjang W, Jiraviriyakul A, Ruchirawat S, Chatwichien J. LC-MS/MS metabolomics-facilitated identification of the active compounds responsible for anti-allergic activity of the ethanol extract of Xenostegia tridentata. *PLoS One* (2022) 17:e0265505. doi: 10.1371/journal.pone.0265505

14. De Paepe E, Van Gijseghem L, De Spiegeleer M, Cox E, Vanhaecke L. A Systematic Review of Metabolic Alterations Underlying IgE-Mediated Food Allergy in Children. *Mol Nutr Food Res* (2021) 65:e2100536. doi: 10.1002/mnfr.202100536

15. Ogulur I, Pat Y, Ardicli O, Barletta E, Cevhertas L, Fernandez-Santamaria R, Huang M, Bel Imam M, Koch J, Ma S, et al. Advances and highlights in biomarkers of allergic diseases. *Allergy* (2021) 76:3659–3686. doi: 10.1111/all.15089

16. Agopian J, Da Costa Q, Nguyen QV, Scorrano G, Kousteridou P, Yuan M, Chelbi R, Goubard A, Castellano R, Maurizio J, et al. GlcNAc is a mast-cell chromatin-remodeling oncometabolite that promotes systemic mastocytosis aggressiveness. *Blood* (2021) 138:1590–1602. doi: 10.1182/blood.2020008948

17. Joseph A, Simonaggio A, Stoclin A, Vieillard-Baron A, Geri G, Oudard S, Michot J-M, Lambotte O, Azoulay E, Lemiale V. Immune-related adverse events: a retrospective look into the future of oncology in the intensive care unit. *Ann Intensive Care* (2020) 10:143. doi: 10.1186/s13613-020-00761-w

18. Schmitt C, Bastek T, Stelzer A, Schneider T, Fischer M, Hackl T. Detection of Peanut Adulteration in Food Samples by Nuclear Magnetic Resonance Spectroscopy. *J Agric Food Chem* (2020) 68:14364–14373. doi: 10.1021/acs.jafc.0c01999

19. Doña I, Pérez-Sánchez N, Eguiluz-Gracia I, Muñoz-Cano R, Bartra J, Torres MJ, Cornejo-García JA. Progress in understanding hypersensitivity reactions to nonsteroidal anti-inflammatory drugs. *Allergy* (2020) 75:561–575. doi: 10.1111/all.14032

20. Beck SC, Wilding T, Buka RJ, Baretto RL, Huissoon AP, Krishna MT. Biomarkers in Human Anaphylaxis: A Critical Appraisal of Current Evidence and Perspectives. *Front Immunol* (2019) 10:494. doi: 10.3389/fimmu.2019.00494

21. Murakami M, Miki Y, Sato H, Murase R, Taketomi Y, Yamamoto K. Group IID, IIE, IIF and III secreted phospholipase A2s. *Biochim Biophys Acta Mol Cell Biol Lipids* (2019) 1864:803–818. doi: 10.1016/j.bbalip.2018.08.014

22. Dinis-Oliveira RJ. Metabolic Profiles of Propofol and Fospropofol: Clinical and Forensic Interpretative Aspects. *Biomed Res Int* (2018) 2018:6852857. doi: 10.1155/2018/6852857

23. Khoo LW, Audrey Kow SF, Maulidiani M, Lee MT, Tan CP, Shaari K, Tham CL, Abas F. Plasma and urine metabolite profiling reveals the protective effect of Clinacanthus nutans in an ovalbumin-induced anaphylaxis model: 1H-NMR metabolomics approach. *J Pharm Biomed Anal* (2018) 158:438–450. doi: 10.1016/j.jpba.2018.06.038

24. Shimanaka Y, Kono N, Taketomi Y, Arita M, Okayama Y, Tanaka Y, Nishito Y, Mochizuki T, Kusuhara H, Adibekian A, et al. Omega-3 fatty acid epoxides are autocrine mediators that control the magnitude of IgE-mediated mast cell activation. *Nat Med* (2017) 23:1287–1297. doi: 10.1038/nm.4417

25. Steinke JW, Pochan SL, James HR, Platts-Mills TAE, Commins SP. Altered metabolic profile in patients with IgE to galactose-alpha-1,3-galactose following in vivo food challenge. *J Allergy Clin Immunol* (2016) 138:1465-1467.e8. doi: 10.1016/j.jaci.2016.05.021

26. Kong J, Chalcraft K, Mandur TS, Jimenez-Saiz R, Walker TD, Goncharova S, Gordon ME, Naji L, Flader K, Larché M, et al. Comprehensive metabolomics identifies the alarmin uric acid as a critical signal for the induction of peanut allergy. *Allergy* (2015) 70:495–505. doi: 10.1111/all.12579

27. Pettersson J, Karlsson PC, Choi YH, Verpoorte R, Rafter JJ, Bohlin L. NMR metabolomic analysis of fecal water from subjects on a vegetarian diet. *Biol Pharm Bull* (2008) 31:1192–1198. doi: 10.1248/bpb.31.1192
